# Psychotherapy 2.0 - Application context and effectiveness of sensor technology in psychotherapy with children and adolescents: A systematic review

**DOI:** 10.1016/j.invent.2024.100785

**Published:** 2024-11-06

**Authors:** Annika K. Alt, Anja Pascher, Lennart Seizer, Marlene von Fraunberg, Annette Conzelmann, Tobias J. Renner

**Affiliations:** a Department of Child and Adolescent Psychiatry, Psychosomatics and Psychotherapy, University Hospital of Psychiatry and Psychotherapy, Tübingen, Germany; b DZPG (German Center for Mental Health), partner site Tübingen, Germany; c PFH – Private University of Applied Sciences, Department of Psychology (Clinical Psychology II), Göttingen, Germany

## Abstract

**Background:**

E-mental health applications have been increasingly used in the psychotherapeutic care of patients for several years. State-of-the-art sensor technology could be used to determine digital biomarkers for the diagnosis of mental disorders. Furthermore, by integrating sensors into treatment, relevant contextual information (e.g. field of gaze, stress levels) could be made transparent and improve the treatment of people with mental disorders. An overview of studies on this approach would be useful to provide information about the current status quo.

**Methods:**

A systematic review of the use of sensor technology in psychotherapy for children and adolescents was conducted with the aim of investigating the use and effectiveness of sensory technology in psychotherapy treatment. Five databases were searched for studies ranging from 2000 to 2023. The study was registered by PROSPERO (CRD42023374219), conducted according to Cochrane recommendations and used the PRISMA reporting guideline.

**Results:**

Of the 38.560 hits in the search, only 10 publications met the inclusion criteria, including 3 RCTs and 7 pilot studies with a total of 257 subjects. The study population consisted of children and adolescents aged 6 to 19 years with mental disorders such as OCD, anxiety disorders, PTSD, anorexia nervosa and autistic behavior. The psychotherapy methods investigated were mostly cognitive behavioral therapy (face-to-face contact) with the treatment method of exposure for various disorders. In most cases, ECG, EDA, eye-tracking and movement sensors were used to measure vital parameters. The heterogeneous studies illustrate a variety of potential useful applications of sensor technology in psychotherapy for adolescents. In some studies, the sensors are implemented in a feasible approach to treatment.

**Conclusion:**

Sensors might enrich psychotherapy in different application contexts.

However, so far there is still a lack of further randomized controlled clinical studies that provide reliable findings on the effectiveness of sensory therapy in psychotherapy for children and adolescents. This could stimulate the embedding of such technologies into psychotherapeutic process.

https://www.crd.york.ac.uk/prospero/display_record.php?ID=CRD42023374219, identifier [CRD42023374219].

## Highlights

• With the help of state-of-the-art sensor technology objective data can be collected.
• This review examined the use and effectiveness of sensor technology in the psychotherapeutic treatment of adolescents.
• Nine studies were included in this systematic review.
• Sensors can be used in psychotherapy for various disorders and application contexts.

## 1 Introduction

Digitalization has opened up new possibilities in the field of psychotherapeutic treatment of patients (Machulska et al., 2016). This development includes digital forms of intervention such as e-mental health apps and video psychotherapy, which can expand existing care options to address the increasing need for therapy of patients and, at the same time, utilize resources effectively (Denecke et al., 2022). Meta-analyses showed, for example, that internet-based psychotherapy via video might be as effective for adults (Batastini et al., 2021; Giovanetti et al., 2022; Matsumoto et al., 2021) and adolescents (Grist et al., 2019; Tang et al., 2018; Vigerland et al., 2016) as traditional face-to-face therapies. Other developments in psychotherapy include the use of wearable sensor technology such as wrist-worn wearables, which make it possible to record patients' biological data such as stress reactions and monitor physiological responses to therapeutic interventions in real time (Reinertsen and Clifford, 2018).

The average age of onset of mental disorder is 14.5 years (Solmi et al., 2022) and early intervention can prevent chronification of the illness (Banaschewski et al., 2022). These advances in digitalization may therefore be particularly important in the treatment of adolescents, as they grow up in a digitalized world and have a high affinity for digital technologies (Hamelmann and Drechsler, 2018). Studies confirm a high level of satisfaction among adolescent patients and their parents with digital treatment approaches (Meininger et al., 2022; von Wirth et al., 2023). In recent decades, research on the use of technology in psychotherapy has increased considerably (Wilhelm and Pfaltz, 2009). Especially in the wake of the Covid-19 pandemic, there has been a rapid increase in the use of digital technologies in psychotherapeutic treatment (Dores et al., 2020; Taylor et al., 2020). To date, however, there has been a lack of systematic evaluations of this novel approach.

Generally, sensor-based measurement techniques require minimal patient engagement and utilize modern, unobtrusive, wearable technology that can be seamlessly integrated into daily life (Abdullah and Choudhury, 2018). These devices, which are often worn on the body, are able to continuously collect data via sensors without interrupting routine activities. In this context, minimal patient engagement refers to application contexts in ambulatory assessments and not to laboratory conditions. They help track various aspects of a person’s physical health and provide initial insights into basic psychological states, such as fluctuations in stress levels and composure (Berrocal et al., 2020). A recent review highlighted the existence of 438 unique wearable devices, typically worn on the wrist, head, chest, ear or arm (Wac, 2018). These devices are designed to monitor various physiological indicators, including heart rate (HR) (Fuller et al., 2020), heart rate variability (HRV) (Stangl and Riedl, 2022), body temperature (Dolson et al., 2022), respiratory rate (De Fazio et al., 2021), blood oxygen levels (Chan et al., 2022) and skin conductance (Markiewicz et al., 2022). In addition, advances in mobile neuroimaging – such as functional near-infrared spectroscopy (Sun et al., 2018), wearable EEG caps (Boto et al., 2018), and mobile recording of brain activity (Topalovic et al., 2020) facilitate the study of brain activity under real-life conditions. An exceptional category within ambulatory technology is smartphones, which, equipped with numerous sensors, can analyze geolocation, app usage, and social media interactions to infer behavioral patterns and perform digital phenotyping (Montag and Quintana, 2023).

In psychotherapy, wearable sensors have been used in the treatment process for adult patients with various disorders and different functions. With the help of sensors, additional previously invisible information can be obtained, or they can help to substantiate the effectiveness of an intervention (Gomes et al., 2023). In the diagnosis of mental disorders (Faurholt-Jepsen et al., 2015; Llamocca et al., 2022), passive tracking can be used to record specific markers and the severity of a disorder (Jacobson et al., 2020; Moshe et al., 2021; Pedrelli et al., 2020; Tazawa et al., 2020), e.g. by recording the movement behavior of depressed patients through the GPS tracker and the integrated pedometer or the contact behavior of anxiety patients by making phone calls or sending text messages. Sensors can be used to monitor mood states of affective disorders (Monteith et al., 2021; Moshe et al., 2021) or to investigate the relationship between depression and circadian rhythm disruption in patients (Ali et al., 2023) using an actigraphy device. Information resulting from sensors can also be used to assess the risk of relapse in major depressive disorder (Matcham et al., 2022; Zlatintsi et al., 2022) by using digital questionnaires via smartphone in combination with measuring the patient's activity behavior to determine the risk of relapse.

Initial results from pilot studies on the use of sensors in psychotherapy are also available for adolescents – however, proof of efficacy is still pending (Sequeira et al., 2020; Welch et al., 2022).

In general, the use of sensors in adolescent psychotherapy offers various advantages, such as objective data collection – because, in contrast to subjective patient reports, sensors objectively measure biological and physiological reactions (Pakhomov et al., 2020). In addition, it is possible to identify symptoms of mental illness at an early stage, such as stress symptoms, and to continuously monitor behavioral data and vital signs (such as heart rate, skin conductance, movements) in real time (Rottstädt et al., 2024). The use of sensors provides detailed patient data for children and adolescents, and the sensor data can be used to adapt the therapy to the needs of the patient.

In addition to the advantages that sensors bring to psychotherapy, there are also disadvantages, such as data protection issues and uncertainties on the part of patients regarding the privacy and security of their data (Karcher and Presser, 2018). Furthermore, technologies are not without error and can also provide incorrect or misleading data due to technical faults (Bläsing, 2017). Patients or their parents could also feel that they are being monitored and that they are completely transparent. Not everyone is interested in technology and not everyone has the same level of technical affinity, so some patients and therapists could reject treatment with sensors (Krömer and Zwillich, 2014).

Sensors have been used in adolescents in a similar way to adults for different mental disorders and with different goals: Using a wearable sensor attached to the foot, the movements and movement complexity of young children with specific autistic characteristics (Wilson et al., 2021) can be recorded and compared with autistic-specific movement patterns. In adolescent patients, a pilot study has investigated whether physiological data and obsessive-compulsive symptoms (Mullick et al., 2022) or depression symptoms (Lønfeldt et al., 2022) can be collected to predict the severity of the disorder using a machine learning approach. A planned review on obese adolescents (Kassim et al., 2022) aims to investigate sensor-based accelerometers that increase physical activity and reduce obesity in patients.

Eye-tracking glasses have already been used in a large number of studies with adult and adolescent patients to determine physiological markers. In addition to studies on attention distortion in anorexia nervosa with the aim of measuring the eye fixation duration presentation of food stimuli (Kang and Chai, 2022; Meregalli et al., 2023; Puttevils et al., 2023), the distorted perception of one's own body image (Hartmann et al., 2020), the increased concentration on the body instead of faces (Sfärlea et al., 2023) and the avoidance of eye contact (Nuding et al., 2024) in comparison to non-anorexic patients, visual adherence to certain foods in binge eating disorder (Sperling et al., 2017) could be demonstrated with the help of eye-tracking glasses.

HR and HRV are also often investigated as a bio signal in mental disorders (Mouga et al., 2021). Previous studies have used ECG sensors to determine biological correlates of mental disorders in adults and adolescents, such as in depression (Chen et al., 2022; Hartmann et al., 2019; Vloet et al., 2019), anxiety disorder (Chalmers et al., 2014), schizophrenia and anorexia (Galetta et al., 2003), and Autism (Bazelmans et al., 2019; Haigh et al., 2021). For example, Chalmers et al. (2014) specify in a meta-analysis that patients with an anxiety disorder have a lower HRV than healthy control subjects (Chalmers et al., 2014). Similar results were concluded by Hartmann et al. (2019) in a study on heart rate variability in depression (Hartmann et al., 2019). The research team found that there were differences in HRV between depressed and healthy subjects and that the severity of depression symptoms correlated with changes in HRV parameters (Hartmann et al., 2019).

Further, ECG may be used to predict the risk of suicide in various psychiatric disorders (Lee et al., 2021), as a marker for stress reactions (Kim et al., 2018), or as a prognosis for treatment success in PTSD (Soder et al., 2019).

Moreover, sensors can also be actively used as a component in the psychotherapeutic treatment of patients and make previously invisible processes visible. In previous studies, cognitive behavioral therapy methods were used to record the heart rate and heart rate variability of patients with PTSD (Saraiya et al., 2022), fear of flying (Busscher et al., 2013), obsessive-compulsive disorder (Duncko and Veale, 2016) and agoraphobia (Mumm et al., 2019) in parallel to psychotherapy with exposures in order to investigate physiological arousal during treatment. Raghav et al. (2016) combined the performance of Virtual Reality Exposure Therapy (VRET) in patients with dental phobia with the recording of heart rate to investigate the subjective tension of patients during virtual exposure (Raghav et al., 2016).

The use of sensory technology in psychotherapy seems particularly useful for adolescents, since the life phase of children and adolescents up to early adulthood is characterized by constant changes in cognitions, emotions and social skills (Lohaus, 2018). Due to their age and associated level of development, as well as their linguistic abilities, some children and adolescents are unable to provide valid information about their level of tension or the intensity of their stress symptoms, so that the addition of objective data, such as sensor data, can be useful (Smith and Hudson, 2013).

Despite the ongoing digitalization in psychotherapy combined with the use of state-of-the-art sensor technology and promising results in adults, research on sensor-based psychotherapy, in which sensors are actively integrated into the psychotherapeutic process, is limited in children and adolescents. In this systematic review, we summarize the current state of research on this topic and provide an overview of applied technologies, methods, results, and the effectiveness of previous studies to facilitate the continuation of research in this area.

### 1.1 Questions and objectives

The aim of this systemic review is to examine the current state of research on the use and effectiveness of sensory therapy in psychotherapy for children and adolescents. Existing research findings will be systematically analyzed and processed based on the following questions: Which (1) type of sensory technology is used with which (2) goal, for which (3) disorder, for which (4) age group of patients? How (5) effective is sensor-based psychotherapy?

## 2 Methods

### 2.1 Protocol and registration

The systematic review was registered with PROSPERO under the registration number CRD42023374219 (Alt et al., 2023). To ensure the quality of the review, the Preferred Reporting Items For Systematic Reviews and Meta-Analysis (PRISMA-P) and a PRISMA flowchart (Moher et al., 2009) were used. The complete checklist for this review can be found in the Appendix.

### 2.2 Selection criteria

The PICO(S) scheme (Aromataris and Riitano, 2014) was used to define the research question and the inclusion and exclusion criteria. All kinds of studies were included that (1) investigated children and/or adolescents with an average age ≤18 years, (2) had a mental disorder diagnosed according to DSM-V (American Psychiatric Association, 2013) or ICD-10 (Dilling and Mombour, 1991), (3) conducted a psychotherapeutic guideline treatment in face-to-face contact or online via video, or (4) used a sensor worn on the body and integrated into the therapy process. Hereby a sensor was defined as any type of technical device that measures vital signs or movements. The outcome (5) was defined as the use, scope and aim of the sensor application in psychotherapy in order to compare the different studies with each other, provided that proof of efficacy was available. The included studies had to be available in (6) full text and in (7) English or German.

Studies were excluded if (1) the patients were on average ≥18 years old, (2) the sensor was only used for pre- and post-treatment measurements as a part of diagnostic process only, (3) or the sensor was used selectively for a specific exercise, e.g. a stress test with a sensor, was conducted, or if a single intervention, e.g. exposure to psychotherapy, was assessed without the intervention being embedded in psychotherapy. The exclusion criteria were chosen because a solid research base on the use of sensors for pre- and post-measurement as well as biofeedback and neurofeedback are already available. We also excluded studies that (4) included biofeedback or neurofeedback or studies that (5) only used virtual reality as a treatment method without an additional sensor. Articles for which full text could not be obtained and other reviews were also excluded.

### 2.3 Systematic literature and coding

The systematic literature search was conducted using a predefined search strategy, in the months of November and December 2023, in the electronic databases PubMed (MEDLINE), Web of Science (Ovid), Cochrane Library, APA PsycInfo (EBSCOhost) and PSYNDEX (EBSCOhost). Each database was searched for articles published between 2000 and 2023. This search period was chosen because the first portable devices, such as fitness trackers and other wearables for measuring heart rate or skin conductivity, were developed around the turn of the millennium and sensor technologies for mental illnesses have been increasingly clinically investigated since 2010. The search strategy of the review consisted of 3 search components. We searched for a psychotherapeutic treatment, a mental disorder, and a sensor. For the search, generic terms of the search components were used. In addition to the term "psychotherapy", similar terms such as treatment, CBT, and iCBT were also included. For "mental illness", specific mental disorders such as anxiety or obsessive-compulsive disorder, post-traumatic stress disorder, ADHD, etc. were also searched for. The "sensor" used was searched for using terms such as sensor-based, sensor-supported, technology, wearable, or by types of sensors in the form of ECG chest straps, eye-tracking glasses, EDA sensors, motion sensors, or by the variable to be measured such as stress, tension or movement. The search terms were searched in titles (ti), abstracts (ab), all fields (af) and MeSh terms. Boolean operators such as "and" and "or" were used to combine the search terms. “Grey literature” was searched for using various access methods (Godin et al., 2015) and included to avoid a selection bias. In addition to the manual search via the Google Scholar search engine, the authors of the included papers were contacted by e-mail to obtain further unpublished literature, and additional searches were carried out in literature databases for grey literature. Detailed documentation of the literature search strategies used in the databases can be found in Table 1 in the Appendix.

### 2.4 Study selection and data extraction/coding

All search results were managed centrally in a library in the literature management program EndNoteTM 20 (The EndNote Team, 2013). After checking the duplicates, titles and abstracts were screened for predefined inclusion criteria and the availability of the full texts was ensured (Bramer et al., 2017). The articles potentially selected for the systematic review were uploaded to Rayyan (Ouzzani et al., 2016) and assessed by two independent reviewers A.K.A. and A.P.

**Fig. 1:**
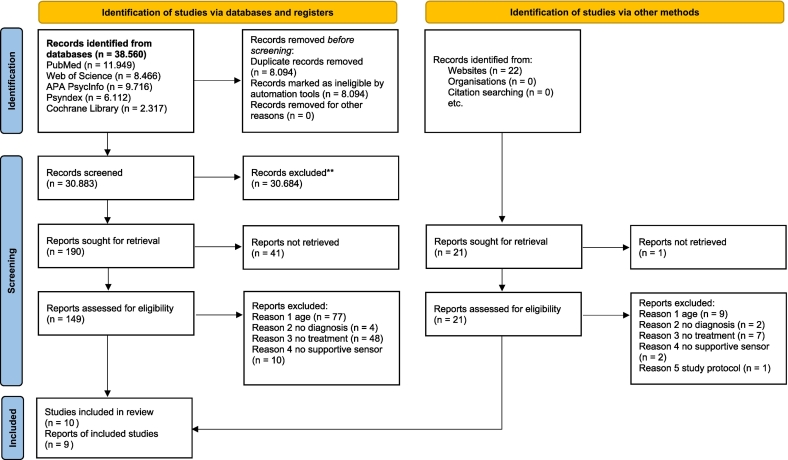
Prisma Checklist

The agreement in terms of interrater reliability was 98.7 %. All studies that did not meet the inclusion criteria based on the assessments of the two reviewers were excluded. In case of discrepancies, the studies in question were discussed and, if necessary, a third reviewer was involved in the discussion. A flow diagram of the literature search is given in Fig. 1.

The data from the studies were transferred to a web-based extraction form. The aim of the extraction was to structure the data and ensure the consistency of the extraction process. Where available, information such as publication details (study name, authors, year), study design and methods (population, number of therapy hours, sensor used), sample characteristics (number of subjects, age, gender), aim of the sensor used and outcome were extracted.

### 2.5 Risk of bias and quality of evidence assessment

The Cochrane tool for assessing risk of bias in randomized trials, RoB2, was used to assess the quality of the included randomized clinical trials (Sterne et al., 2019). Biases in sample allocation (selection bias), treatment (performance bias), recording of results (detection bias), group differences (attrition bias) and selective reporting of positive trial results (reporting bias) were assessed in five different domains (Buchberger et al., 2014).

For single-arm, non-randomized studies, such as feasibility studies, there is currently no standardized assessment tool for evaluating the quality of the risk of bias (Cochrane Deutschland, 2016). For this reason, the included feasibility studies were analyzed descriptively based on the Cochrane Collaboration's tool for non-randomized studies, ROBINS-I (Sterne et al., 2016). Biases reported included selection of study participants, classification of interventions, deviation from intended interventions, missing data from, e.g., initially included individuals, measurement of outcome data and selection of reported outcomes. Biases are assessed for RCTs with low, high or unclear risk. Robvis (McGuinness and Higgins, 2021) was used to illustrate the assessment of risk of bias of RCTs. The quality assessment of the studies was done by two persons A.K.A and L.S., independently of each other.

### 2.6 Data synthesis

Due to the heterogeneity of the included studies, such as method, design, population, sensor used and missing information, such as effect sizes or variance information, which would be required for a meta-analysis, no meta-analytical aggregation of the data was performed. Instead, a systematic narrative data synthesis was chosen as the form of analysis (Rodgers et al., 2009). The aim of narrative synthesis is to systematically review and summarize the results of multiple studies, develop a theory, produce a preliminary synthesis of the results, examine the relationship of the data to each other, and assess the robustness of the synthesis using words and text (Ryan, 2013). The coded text parts were first organized into main categories (Authors, Country, Disease, Sample, Intervention, Sensor, Aim, Result). Subsequently, the extracted data were analyzed in relation to each other in the context of the study design, with a focus on the sensor-based therapy intervention (Centre for Reviews and Dissemination, 2009). The included studies, the analyzed variables and main categories are presented in Table 2.

**Table 2:** Categorization of the studies

**Authors**	**Country**	**Diagnoses**	**Sample**	**Intervention**		**Sensor Types**	**Sensor Functions**	**Aim**	**Effect sized Result**
				**Sessions**	**Therapy content**				
Ascione et al., 2023	SPA	Anorexia Nervosa	n = 23 12-17.11 years mean = 15.30 SD = 1.29 male = 0 female = 23	1 session (duration of 10-15 min.)	Exposure	Eye Tracker, VR	Treatment Efficacy	Investigating the effectiveness of a single session of a new body-based AB modification task (ABMT) that combines virtual reality with eye-tracking to reduce body-related attention.	The duration of fixation of weight- related body parts was significantly reduced compared to the measurement before the one-hour training session (*t*(23) = 1.86 *p* = .04, *d* = .45) and body satisfaction (*t*(23) = 1.89, *p* = .04, *d* = .39) increased.
Kahlon et al., 2019	NOR	Public Speaking Anxiety	n = 27 13-16 years mean = 14.22 SD = 0.64 male = 6 female = 21	1 session (duration of 90 min.)	VR-Exposure	ECG	Treatment Efficacy	Investigation of moderators of treatment and heart rate during the VR-based exposure intervention.	(VRET) has proven to be an effective tool for the treatment of adolescents with speech anxiety and the sensors used for physiological data show a slight increase in HR during exposures. Significant decrease in PSA symptoms (*p* < .01, *d* = 1.53) and consistent improvement at follow-up.
López-Florit et al., 2021	SPA	Autism	n = 16 6-18 years mean = 12.5 SD = 2.99 male = 16 female = 0	26 sessions	Turn-Taking	ECG of the therapist	Additional information	Examination of the therapist-patient communication process and turn-taking based on the patient's inter-personal and social skills. Recording the therapist's HRV to identify stress.	The communicative intention of the patients (more turn-taking events) is determined by their social and intellectual (IQ) abilities (*r* = .53, *p* = .05). Speaker change was associated with the patient's emotional and behavioral difficulties (self-report antisocial behavior, *r* = .70, *p* = .05). Therapists' HRV increased when the session required higher synchrony (χ2 7.09; df 2; *p* = .05).
Thierfelder et al., 2022	GER	OCD	n = 5 13-17 years mean = 15.2 male = 4 female = 1	Several sessions	Exposure	ECG, Eye Tracker, Motion sensors	Additional information	Verification of a sensor system for measuring stress and anxiety in adolescent compulsive patients.	The increasing stress was detectable both in the ECG and the other sensors. The ECG analysis showed an increasing heart rate and a decreasing HRV. No statistical data is reported.
Zimmermann et al., 2021	AUS	Borderline Disorder	n = 16 (32) 13–19 years mean = 16.6 SD = 1.5 male = 0 female = 16	356 sessions, 16 completed psycho-therapies	AIT	ECG, EDA	Additional information	Investigation of movement synchrony in female adolescents with borderline personality disorder.	Higher levels of movement synchrony in therapy sessions compared to pseudo-interactions (*p* = .01, *d* = .85) and correlation of movement synchrony with better therapy outcome (β = -0.43, *p* = .02, CI: -0.02 –0.00).
Lipschutz et al., 2017	USA	PTSD	n = 48 7-13 years mean = 9.7 SD = 1.9 male = 28 female = 20	12 sessions	Exposure	ECG	Additional information	Examination of RSA patterns in children and gender differences with PTSD symptoms and their change after treatment.	Time was significant (F(1,46) = 74.93, *p* = .01), indicating that PTSD symptoms were significantly reduced after CBT. The decrease in PTSD symptoms is not related to resting RSA before treatment. (F(1, 46) = .53, *p*= .47)
McCormack et al., 2020	USA	Anxiety	n = 18 7-14 years mean = 11.38 SD = 1.66 male = 9 female = 9	12 sessions	Exposure	EDA	Additional information	Relation between EDA and subjective self-assessment of anxiety and prediction of treatment success.	No significant association of self-reported anxiety and physiological arousal. Strong correlation between increased sympathetic arousal and poorer response after treatment (β = 0.32, *t*(32) = 2.25, *p* = .03, CI: 0.09-1.73). Physiological arousal as the strongest predictor of treatment response (*r* = .50, *p* = .03).
Shenket al. 2022	USA	PTSD	n = 33 6-17 years mean = 11.79 SD = 3.08 male = 11 female = 22	8 sessions	Exposure	ECG	Treatment Efficacy	Investigation of the effectiveness and effects of animal-assisted therapy as an adjunct to trauma-focused CBT treatment for PTSD on RSA compared to standardized trauma-focused CBT.	Significant changes in the decrease RSA during trauma-focused CBT (δ001 = .08, *p* = .85), regardless of treatment group. Animal-assisted therapy had no effect on patients' RSA.
Voss et al., 2019	USA	Autism	n = 71 6-12 years mean = 8.38 SD = 2.46 male = 63 female = 8	6-minute sessions (4 times a week for 20 weeks)	Facial Engagement, Emotion Recognition	Superpower Glass	Treatment Efficacy	Improving and training the recognition of emotions and faces using AI-based Google glasses.	The intervention increased the recognition of emotions and faces (VABS-II Socialization *r* = .9, *p* = .01), but there was no significant predictor of responding to treatment for the other endpoints between the cohorts (NEPSY-II *r* = .08, *p* = .93).

Abbreviations: AMBT, Attentional Bias Modification Task; AI, Artificial Intelligence; ß, Beta Coefficient; CBT, Cognitive Behavioral Therapy; χ2, Chi-square test ; CI, Confidence Interval; δ, delta ; d, Cohens d; df, Degrees of Freedom; ECG, Electrocardiogram; EDA, Electrodermal Activity; F, Analysis of Variance; HR, Heart Rate; HRV, Heart Rate Variability; IQ, Intelligence Quotient; NEPSY-II, Developmental Neuropsychological Assessment; r, Regression Analysis; RSA, Respiratory Sinus Arrhythmia; p, P Value; PTSD, Post Traumatic Stress Disorder; SD, Standard Deviation; t, t-Test; VABS-II Socialization, Vineland Adaptive Behavioral Scales; VR, Virtual Reality ; VRET, Virtual Reality Exposure Therapy

## 3 Results

### 3.1 Study selection

A total of 38.560 articles were identified in the initial search. After excluding duplicates, 30.883 studies were reviewed for inclusion in the study. 30.684 articles were excluded because they did not meet the inclusion criteria. 149 full-text articles were analyzed in more detail, resulting in 10 studies being included in the systematic review. Two articles by Thierfelder et al. (2022) and Primbs et al. (2022) reported on the same study, so that only one of the two studies was included in the analysis. The final number of included studies was 9 articles. A supplementary search of gray literature found one study protocol, but it was excluded due to a lack of results. See Fig. 1 for the PRISMA flow diagram.

### 3.2 Characteristics of the studies included in the review

All characteristics of the included studies, divided into different categories, are shown in Table 2. The studies were published between 2017 and 2023 and the majority were conducted in Europe (Ascione et al., 2023; Kahlon et al., 2019; López-Florit et al., 2021; Thierfelder et al., 2022; Zimmermann et al., 2021) and the USA (Lipschutz et al., 2017; McCormack et al., 2020; Shenk et al., 2022; Voss et al., 2019). Most of the studies were pilot or feasibility studies, without randomization (Ascione et al., 2023; Kahlon et al., 2019; López-Florit et al., 2021; McCormack et al., 2020; Thierfelder et al., 2022; Zimmermann et al., 2021). Only 3 of the included studies were RCTs (Lipschutz et al., 2017; Shenk et al., 2022; Voss et al., 2019).

### 3.3 Study population

A total of 257 subjects (53% male) aged between 6 and 19 years with an average age of 12.79 years took part in the nine included and completed studies. The sample size of the studies varied between 5 and 71 participants.

### 3.4 Diagnosis

Two studies each treated children and adolescents with Autism (López-Florit et al., 2021; Voss et al., 2019), anxiety disorder (Kahlon et al., 2019; McCormack et al., 2020) and a post-traumatic stress disorder (Lipschutz et al., 2017; Shenk et al., 2022). One study each described the treatment of children and adolescents with anorexia nervosa (Ascione et al., 2023), borderline personality disorder (Zimmermann et al., 2021) and obsessive-compulsive disorder (Thierfelder et al., 2022).

### 3.5 Intervention/treatment/number of sessions

In 7 of the 9 studies, the treatment of children and adolescents took place in face-to-face contact (Ascione et al., 2023; Kahlon et al., 2019; Lipschutz et al., 2017; McCormack et al., 2020; Shenk et al., 2022; Voss et al., 2019; Zimmermann et al., 2021). The number of sessions ranged from a single session (Ascione et al., 2023; Kahlon et al., 2019) to 26 sessions, a short-term therapy (López-Florit et al., 2021). The treatments focused on behavioral interventions in which the sensor was used during one or more exposure exercises (Ascione et al., 2023; Kahlon et al., 2019; Lipschutz et al., 2017; McCormack et al., 2020; Shenk et al., 2022; Thierfelder et al., 2022; Voss et al., 2019).

### 3.6 Sensors used/aim/sensor functions

In the psychotherapy sessions, ECG sensors (Kahlon et al., 2019; López-Florit et al., 2021; McCormack et al., 2020; Shenk et al., 2022) were predominantly used to measure HR and HRV in patients or therapists, EDA skin sensors (McCormack et al., 2020) to measure electrodermal activity, movement sensors (Zimmermann et al., 2021) to measure movement synchrony or to recognize movement patterns, and VR and eye-tracking glasses (Ascione et al., 2023) to identify gaze patterns. In one study each, a multimodal sensor set consisting of various sensors (ECG, eye-tracking glasses, motion sensors) (Thierfelder et al., 2022) was used to measure and recognize stress reactions and an AI Google Glass (Voss et al., 2019) was used to train emotion recognition. The sensors were used for different purposes: in some studies, they served to demonstrate the effectiveness of the related intervention (Ascione et al., 2023; Kahlon et al., 2019; Shenk et al., 2022; Voss et al., 2019) while in other studies, the sensors provided additional information (Lipschutz et al., 2017; López-Florit et al., 2021; McCormack et al., 2020; Thierfelder et al., 2022; Zimmermann et al., 2021), such as the increase in heart rate in exposure sessions in obsessive-compulsive patients (Thierfelder et al., 2022).

## 4 Results

The sensors provided additional information from either the patient or therapist during the therapy sessions. López-Florit et al. (2021) was able to objectively examine the change of speaker between patients and therapists with the help of sensors and found that the communicative intention of the patients is determined by their social and intellectual abilities (*r* = .53, *p* = .05), and that turn-taking was related to the patient's emotional and behavioral difficulties (self-reported antisocial behavior, *r* = .70, *p* = .05). Therapists' stress levels, as measured by HRV, increased when the session required higher synchrony (χ2 7.09; df 2; *p* = .05). Thierfelder et al. (2022) demonstrated that patients experience stress during an exposure session and record it using ECG sensors as well as other sensors. The ECG analysis showed an increasing heart rate and decreasing HRV, indicating high levels of tension. Higher movement synchrony in therapy sessions compared to pseudo-interactions (*p* = .01, d = .85) could be substantiated by Zimmermann et al. (2021) in female borderline patients and found that movement synchrony correlates with better therapy outcome (β = -0.43, *p* = .02, CI: -0.02 –0.00). McCormack et al. (2020) found no significant association between self-reported anxiety and physiological arousal in adolescents. As a result, he found that increased sympathetic arousal was associated with a poorer response to treatment (β = 0.32, *t*(32) = 2.25, *p* = .03, CI: 0.09–1.73). The physiological arousal of the patients was the strongest predictor of the response to treatment (*r* = .50, *p* = .03). Time appears to be a significant factor in the treatment of PTSD according to Lipschutz et al. (2017) (F(1,46) = 74.93, *p* = .01), since PTSD symptoms were significantly reduced after CBT. No association was found between pre-treatment resting RSA and the reduction in PTSD symptoms (F(1, 46) = .53, *p* = .47).

With the help of sensors, some studies have also been able to examine the reduction of symptoms and the effectiveness of the treatment. Kahlon et al. (2019) used virtual reality exposure therapy in adolescents with speaking anxiety and was able to show a slight increase in heart rate during exposure and a significant decrease in symptoms (*p* < .01, *d* = 1.53) using the sensors. Shenk et al. (2022) also found no change in RSA during trauma-focused CBT (δ001 = .08, *p* = .85), regardless of treatment group. Ascione et al. (2023) examined the duration of fixation on weight-related body parts in patients with anorexia nervosa and found that the ABMT task significantly reduced fixation time on weight-related body parts (t(23) = 1 .86, *p* = .04, *d* = .45) and body satisfaction increased (*t*(23) = 1.89, *p* = .04, *d* = 0.39). Voss et al. (2019) indicated that Superpower Glass was suitable as an intervention for training emotion and face recognition (VABS-II socialization *r* = .9, *p* = .01). No predictor of treatment response between cohorts could be identified in the other outcome measures (NEPSY-II *r* = .08, *p* = .93).

### 4.1 Assessment of risk of bias in the studies included in the review

**Fig. 2:**
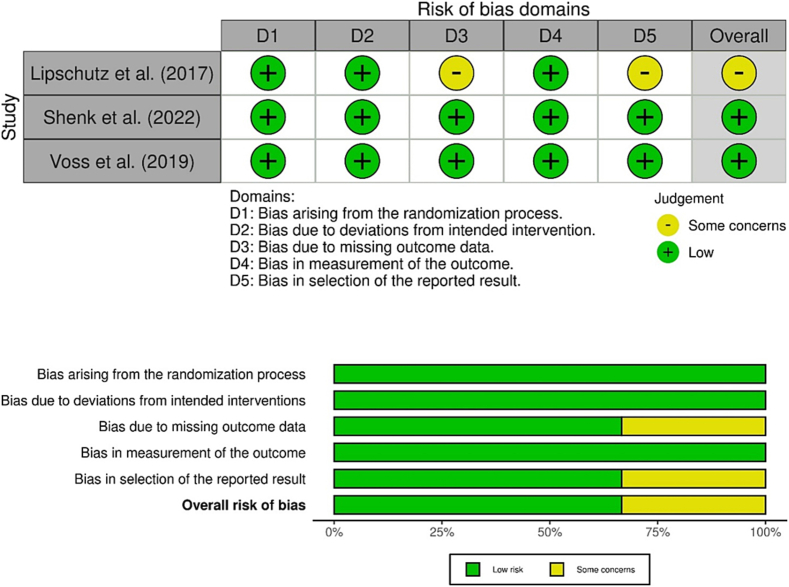
Cochrane Risk of Bias (ROB-2).

The results of the quality assessment of the three studies included in this systematic review with regard to risk of bias are shown in Fig. 2. For the remaining six included non-randomized feasibility studies, the risk of bias is described descriptively due to the lack of standardized assessment tools.

Overall, the RCTs (Lipschutz et al., 2017; Shenk et al., 2022; Voss et al., 2019) were evaluated as having a low to medium risk of bias. Subjects were selected in all studies using predefined inclusion and exclusion criteria (Lipschutz et al., 2017; Shenk et al., 2022; Voss et al., 2019). The randomization of participants was blinded in the RCTs, so that a low bias in the selection, inclusion of participants and allocation to the intervention can be assumed (Lipschutz et al., 2017; Shenk et al., 2022; Voss et al., 2019). No deviations from the planned intervention of the subjects were described during the treatment in the studies (Lipschutz et al., 2017; Shenk et al., 2022; Voss et al., 2019). The results were fully reported in two of three studies (Shenk et al., 2022; Voss et al., 2019) and were not biased by missing data, as the missing data were accounted for by an intention-to-treat procedure. In the randomized controlled trial by Lipschutz et al. (2017), dropouts occurred during treatment; the reasons for dropouts during ongoing treatment were not reported and not included in the results of the study.

In the included pilot and feasibility studies (Ascione et al., 2023; Kahlon et al., 2019; López-Florit et al., 2021; McCormack et al., 2020; Thierfelder et al., 2022; Zimmermann et al., 2021), the overall risk of bias of the reported study results must be considered as high, as there was no control or comparison group for the proof of efficacy.

In almost all studies, bias cannot be ruled out in the selection of study participants because, for example, only female (Ascione et al., 2023) or male subjects (López-Florit et al., 2021) were included or selective recruitment in school classes was used (Kahlon et al., 2019) or subjects were selected based on the progress of exposure treatment (McCormack et al., 2020). Allocation to the respective intervention in the study took place after inclusion, with the included patients receiving the same treatment in all pilot studies (Ascione et al., 2023; López-Florit et al., 2021; Thierfelder et al., 2022; Zimmermann et al., 2021). In one study the intervention measure was adapted to the needs of the patients and extended during treatment, so that some patients received a longer intervention than others (Kahlon et al., 2019; McCormack et al., 2020); a bias in the intervention can be assumed here. Outcome measurement in the feasibility studies (Ascione et al., 2023; Kahlon et al., 2019; López-Florit et al., 2021; McCormack et al., 2020; Zimmermann et al., 2021) was based on predefined criteria. In some studies, only part of the data was included in the results, so that selective results were reported without the inclusion of dropouts (Kahlon et al., 2019; López-Florit et al., 2021; Thierfelder et al., 2022; Zimmermann et al., 2021). One pilot study (Thierfelder et al., 2022) did not include relevant information to assess bias in the different domains.

## 5 Discussion

### 5.1 Principal results

The primary aim of the review was to compile the previous studies on sensor-based psychotherapy with children and adolescents and to obtain an overview of the current state of research on the age range of the investigated patients, the mental disorders treated, the therapy methods and interventions used as well as the effectiveness of the therapy approach.

An important finding was that only a total of 9 studies with a total of ten articles met the inclusion criteria and that even in the "grey literature" only one psychotherapy study with adolescents is designed to integrate some kind of sensor into the therapy process. Due to the (very) limited number of studies, it can be assumed that research in this area is still in its infancy. The number of empirical studies on sensor-supported psychotherapy with adult patients is also rather low. The review by Drissi et al. (2020) on sensory treatment options in psychiatric care also comprised only 12 studies. The number of empirical studies with adult patients also shows that the research field of sensory therapy in psychotherapy is still young. Due to the limited number of studies, it can be assumed that research on this topic is in early stages, both for children and adolescents and for adults.

Our narrative synthesis revealed that sensors are mainly used in psychotherapy studies in the form of ECG and EDA sensors as well as eye-tracking glasses. Sensors were especially used in behavioral therapy interventions such as exposure exercises in disorders, e.g., anorexia nervosa, PTSD, anxiety and obsessive-compulsive disorders. Studies with a depth-psychological-analytical therapy focus are lacking. It is encouraging that sensors can be used in therapy with adolescents for different goals and with different mental disorders, which illustrates the broad spectrum of possible applications of sensors. From the objectification of subjective stress perception in children (McCormack et al., 2020), the measurement of speech alternation (López-Florit et al., 2021), movement synchrony in therapy sessions (Zimmermann et al., 2021), heart rate during virtual exposure (Kahlon et al., 2019), eye movement and fixation duration during body-related exposure (Ascione et al., 2023), the development of a multimodal sensor system for stress detection (Thierfelder et al., 2022), the training of emotion recognition (Voss et al., 2019) through the investigation of gender-specific RSA differences (Lipschutz et al., 2017) or the effects of animal-assisted therapy in comparison to normal CBT (Shenk et al., 2022), sensors convey relevant information and make processes visible. The age range of the adolescents was between 6 and 19 years, with the average age of the studies indicating use by adolescents aged 12.79 years and older. This could be because adolescents aged 12 and above need less support from their parents than children aged below and can use the technical devices more independently.

The effectiveness of sensor-based psychotherapy could not be sufficiently investigated due to the small number of studies. In all 3 RCTs (Lipschutz et al., 2017; Shenk et al., 2022; Voss et al., 2019), the sensors were used with different objectives and produced interesting new results that need to be investigated further in the future. In none of the RCTs (Lipschutz et al., 2017; Shenk et al., 2022; Voss et al., 2019) did the use of sensor technology in therapy lead to a significant improvement compared to the comparison group. However, in two of the RCTs, the sensor was not used to improve the treatment outcome, i.e. to reduce symptoms, but as an indicator for comparative measurement between two cohorts (Lipschutz et al., 2017; Shenk et al., 2022). Neither Shenk et al. (2022) found an improvement in RSA with animal-assisted therapy compared to standard CBT, nor Lipschutz et al. (2017) found any gender-specific change in RSA in adolescent PTSD patients. Our results confirm that research into sensory-based psychotherapy is still in its infancy, but that the approach is feasible in principle. Future research in this novel area is necessary, as the sensors make relevant contextual information visible during treatment, allowing treatment to be adapted more specifically to the patient's needs.

### 5.2 Future work

Future research should investigate the challenges associated with the novel sensor-based therapy approach and how patients experience sensor-based psychotherapy. The provision of additional patient-related information via the internet has both benefits and risks that need to be considered in the future. Patient-related health data is particularly sensitive data, so great attention must be paid to data protection in sensor-based psychotherapy and the protection of patient data (Glenn and Monteith, 2014). Through the use of sensor technology, the patient becomes a kind of "transparent patient" (Mayer et al., 2019) who appears very transparent and visible. In addition, ethical and legal aspects of the use of sensor technology in psychotherapy should be critically discussed and scrutinized (Harris and Birnbaum, 2015). More qualitative research that includes the patient's perspective seems important in order to adapt the intervention based on the experience of those being treated and to clearly highlight the benefits of sensor-based psychotherapy for patients and psychotherapists. Further randomized clinical trials with follow-up are needed to confirm the effectiveness of the sensor-based therapy approach. Once the evidence of efficacy is available, psychotherapists should be better informed and trained about the use of sensors, their application goals and their added value in psychotherapy to ensure successful application (Batterham et al., 2015).

### 5.3 Strengths and limitations

To the best of our knowledge, this is the first review of sensory-based psychotherapy in children and adolescents. It provides a concise overview of the latest research on the use and effectiveness of sensors in psychotherapy. Encouragingly, the review demonstrates that sensors can be used in a variety of disorders and age ranges.

One limitation of the review is the low number and heterogeneity of the included studies, as there is an abundance of sensors, objectives and outcomes. Due to the limited availability of studies on sensor-based psychotherapy in children and adolescents, only three RCTs could be included, which does not clearly demonstrate the effectiveness and benefits of sensors in psychotherapy, as all randomized controlled trials investigated a different disorder with a different sensor, and had a high heterogeneity in effect sizes, which also weakens the validity of the review. A second limitation of the review could be the inclusion of only English- and German-language literature and the selected cohort. It is possible that there are other studies that have been published in a different language and that studies were conducted with adolescents or young adults with a mean age over 18 years. There is a higher number of studies for the use of sensors to measure change, such as through a pre-post design or to determine biomarkers in mental disorders than studies that actively implicate the sensor in treatment.

## 6 Conclusions

The systematic review clearly shows that to date, only a few studies have been carried out on the integration of sensory technology into psychotherapy for children and adolescents. The findings from the use of sensors in psychotherapy sessions are informative and provide objective information through the use of sensors. The additional information can be used, for example, to adjust the level of exposure exercises to the level of habituation measured by the sensors used with anxiety and obsessive-compulsive disorder patients. The better adaptation of psychotherapy to the needs of children and adolescents can promote the success of therapy and possibly prevent the chronification of symptoms at an advanced age. Further research in the field of sensor-based psychotherapy seems necessary to prove the effectiveness of the approach and the benefits of sensors in psychotherapy.

## Abbreviations

AB: Abstract
ABMT: Attentional Bias Modification Task
ADHD: Attention Deficit Hyperactivity Disorder
AF: All fields
AI: Artificial Intelligence
ALL: All Terms
APA: American Psychological Association
ß: Beta Coefficient
CBT: Cognitive Behavioral Therapy
χ2: Chi-square test
CI: Confidence Interval
d: Cohens d
δ: Delta
df: Degrees of Freedom
DSM-V: Diagnostic and Statistical Manual of Mental Disorders
ECG: Electrocardiogram
EDA: Electrodermal Activity
EEG: Electroencephalogram
GPS: Global Positioning System
F: Analysis of Variance
F-2-F: Face-to-Face
HR: Heart Rate
HRV: Heart Rate Variability
iCBT: Internet based Cognitive Behavioral Therapy
ICD-10: International Statistical Classification of Diseases and Related Health Problems
IQ: Intelligence Quotient
ITT: Intention-To-Treat
Mesh: Medical Subject Headings
NEPSY-II: Developmental Neuropsychological Assessment
NRCT: Non-Randomized Controlled Trials
OCD: Obsessive Compulsive Disorder
p: P Value
PICO(S): Population, Intervention, Comparison, Outcome, Study Design
PROSPERO: International Prospective Register for Systematic Reviews
PRISMA: Preferred Reporting Items for Systematic Reviews and Meta-Analyses
PTSD: Post Traumatic Stress Disorder
r: Regression Analysis
ROB 2: Cochrane Risk-Of-Bias Tool for Randomized Trials
ROBINS-I: Cochrane Risk Of Bias In Non-randomized Studies of Interventions
ROBVIS: Risk-of-Bias Visualization Tool
RCT: Randomized Controlled Trial
RSA: Respiratory Sinus Arrhythmia
SD: Standard Deviation
TI: Title
T: T-Test
VABS-II: Vineland Adaptive Behavioral Scales, Category Socialization
Socialization
VR: Virtual Reality
VRET: Virtual Reality Exposure Therapy

## Author contributions

Conceptualization: A.K.A., A.C., T.J.R.; data extraction: A.K.A. and M.F.; validation: A.K.A, A.P. and L.S.; preparation of the original draft, which was critically revised by all authors. All authors read and approved the final manuscript; review/editing/supervision, A.C. and T.J.R.

## Declaration of competing interest

The authors declare that they have no known competing financial interests or personal relationships that could have appeared to influence the work reported in this paper.

## Figures and Tables

**Table 1:** Literature search strategies in databases

Database	Search Strategy
Web of Science	(ALL(Psychotherapy OR cbt OR psychoanalysis OR treatment) OR (eating disorder OR anorexia OR anxiety OR autism OR borderline OR depression OR ocd OR obsessive compulsive disorder OR phobie OR PTSD OR ADHD) AND (wearable OR hrv OR heart rate OR ecg OR sensor OR wearable sensor OR sensor* OR respiratory OR technology OR eye-track* OR sensor assisted) AND/OR (exposure OR synchrony OR movement)
Psyndex	(TITLE-ABS(Psychotherapy) OR (cbt) OR (icbt) AND (sensor*) OR (wearable) OR (ecg) OR (hrv) OR (hr) OR (technology) OR (respiratory) OR (synchrony) OR (movement) OR (eye-track*) OR/AND (anxiety) AND (borderline) AND (autism) AND (anxiety) AND (depression) AND (ocd) AND (anorexia) AND (ptsd) AND (adhd) AND/ OR (exposure)
Cochrane Library	(ALL(Psychotherapy OR cbt OR psychoanalysis OR treatment) OR (eating disorder OR anorexia OR anxiety OR autism OR borderline OR depression OR ocd OR obsessive compulsive disorder OR phobie OR PTSD OR ADHD) AND (wearable OR hrv OR heart rate OR ecg OR sensor OR wearable sensor OR sensor* OR respiratory OR technology OR eye-track* OR or sensor assisted) AND/OR (exposure OR synchrony OR movement)
PubMed	(ALL/Mesh("Psychotherapy” OR "psychotherapy, psychodynamic" OR "psychotherapy, rational emotive" OR "Person-Centered Psychotherapy" OR "Cognitive Behavioral Therapy") OR ("disease" OR "disorder") OR ("anxiety" OR "anxieties" OR "anxiety s") OR ("depressed" OR "depression" OR "depressions" OR "depressive disorder" OR ("depressive" AND "disorder") OR ("depressively" OR "depressiveness" OR "depressives") OR ("obsessive compulsive disorder") OR ("obsessive compulsive" AND "disorder") OR ("obsessive" AND "compulsive" AND "disorder") OR ("feeding and eating disorders" OR ("feeding" AND "eating" AND "disorders") OR ("eating disorder") OR ("sleep wake disorders" OR ("sleep" AND "wake" AND "disorders") OR ("sleep wake disorders") OR ("sleep" AND "disorder") OR ("sleep disorder") OR ("dependence" OR "dependant" OR ""dependants" OR "depended" OR "dependences" OR "dependencies") OR ("dependency" AND "psychological") OR ("psychological dependency" OR "dependency" OR "dependent s" OR "dependently" OR "dependents" OR ""depending" OR "depends") " OR ("anorexia" OR "anorexias") OR ("autisms" OR "autistic disorder" OR ("autistic" AND "disorder") OR ("autism") OR ("substance related" AND "disorders") OR ("substance related disorders") OR ("substance" AND "abuse") OR ("substance abuse") OR ("alcoholism") OR ("alcohol" AND "dependence") OR ("alcohol dependence") OR ("schizophrenias" OR "schizophrenia OR "personality disorders") OR ("personality" AND "disorder") OR ("stress disorders" OR "post traumatic") OR ("stress" AND "disorders" AND "post traumatic") OR ("ptsd") AND (“electrocardiography” OR "electrocardiography, ambulatory" OR "ecg" OR"electrocardiographer" OR "electrocardiographic" OR "electrocardiographical" OR "electrocardiographically" OR "electrocardiographics" OR OR "electrocardiographs") OR ("Heart Rate Determination" OR "Heart Rate" OR "HRV" OR "HR") OR ("Remote Sensing Technology/methods" OR "remote sensing technology/psychology") OR ("sensor" OR "sensors" OR "sensoric" OR "sensorics" OR "sensoring" OR ""sensorization" OR "sensorized") OR/AND ("assist" OR "assists" OR "assistances" OR "assisted" OR ""assisting" OR "assistive" OR “assistants" OR "assistant" OR "helping behavior") OR ("helping" AND "behavior) OR ("based" OR "basing") OR ("wearability" OR "wearable" OR "wearables") OR ("eye tracking" AND "technology") OR ("eye tracking technology" OR "tracking" OR "eye tracking") OR ("eye" OR "eye movement") OR ("body" OR "movement" OR "body movement") OR ("EDA" OR "skin" OR "dermalive" OR "dermal" OR "dermally") OR ("conductivity" OR "electric conductivity" OR "conductivity") OR ("activity" OR "motor activity") OR ("bracelet" OR "bracelets" OR "belt ) OR ("eyeglasses" OR "glasses" OR "glass") OR ("stress" OR "stressed" OR "stresses" OR "stressful" OR "stressfulness" OR "stressing") OR ("motion" OR "motion" OR "motions" OR "motional" OR ("tension" OR "tensional" OR "tensioned" OR "tensioner" OR "tensioners" OR "tensioning" OR "tensions") OR "technology" OR "technologies" OR "technologys")
PsycInfo	(Mesh/TI(Psychotherapy) OR (cbt) OR (icbt) AND (sensor*) OR (wearable) OR (ecg) OR (hrv) OR (hr) OR (technology) OR (respiratory) OR (synchrony) OR (movement) OR (eye-track*) OR/AND (anxiety) AND (borderline) AND (autism) AND (anxiety) AND (depression) AND (ocd) AND (anorexia) AND (ptsd) AND (adhd) AND/ OR (exposure)

## Appendix B Supplementary data

Appendix Prisma-P Checklist Image 1
